# The Effect of “Production during Remediation” of Plants in Cd-Contaminated Soil

**DOI:** 10.3390/toxics10120732

**Published:** 2022-11-26

**Authors:** Lankun Li, Huiqing Chang

**Affiliations:** College of Agriculture, Henan University of Science and Technology, Luoyang 471000, China

**Keywords:** Cd, production during remediation, wheat fields, remediation effects

## Abstract

In order to find suitable plants for “production during remediation” in wheat fields moderately contaminated by cadmium (Cd), five plants—green amaranth, oil sunflower, broomcorn, maize, and waxy maize—were planted in pots to study their enrichment characteristics and remediation effects in Cd-contaminated soil. The results showed that the highest bioaccumulation and translocation factors were greater than 0.5 for oil sunflower, which had the strongest Cd-enrichment ability in Cd-contaminated soil, but its biomass was small, and the Cd content of the grain exceeded the standard (GB2762-2017). The Cd content in the grains of broomcorn, maize, and waxy maize was less than 0.1 mg∙kg^−1^, which is lower than the national food safety standard on limiting pollutants in food (GB2762-2017). Broomcorn accumulated 0.429 mg∙pot^−1^ for Cd, with a Cd-extraction efficiency of 1.73%, which were higher than other plants. Taking the risk-screening values in GB15618-2018 “Soil Environmental Quality Standard” as the target, it will take 80 years to remediate using broomcorn, which has the highest extraction efficiency, based on cultivating remediation plants once per year. However, in view of the scarcity of arable land resources in China and the objective of safe production during remediation, the use of broomcorn can be considered for production during remediation for the given degree of Cd contamination of the soil.

## 1. Introduction

Cadmium (Cd) is one of the most toxic-heavy metals to plants and animals, and long-term consumption of food with excessive Cd can lead to its accumulation and toxicity in the human body, causing diseases, such as cancer, arthritis, and other diseases [1,2]. Thus, the environmental and food security problems caused by Cd pollution are of great concern [3,4]. In the natural state, the content of Cd in the soil ranges from 0.1 to 1.0 mg∙kg^−1^, and human activities, such as mining, smelting, fertilizer application, sludge agriculture, and sewage irrigation, have led to different degrees of Cd contamination in agricultural soils in many regions [5,6].

Although most Cd-contaminated soils are still in a moderate and mild degree, and the contamination is mainly localized, the risk of Cd contamination in the main wheat-producing areas in the north of China has become apparent in recent years, with frequent incidents of “Cd wheat” and other agricultural products with excessive Cd. For example, in 2017, it was reported that the Cd content of wheat in Xinxiang City in Henan Province exceeded the standard (GB2762-2017) by 1.7 to 12.8 times, and the Cd content in the soil of agricultural fields in the suburbs of Xinxiang City exceeded the standard (GB15618-2018). “Cd wheat” has also been detected in some wastewater irrigation areas of Kaifeng and Beijing [7,8]. Therefore, in view of the increasingly scarcity of reserve-arable-land resources in the main wheat-producing areas in northern China, it is particularly important and urgent to carry out remediation and treatment of Cd pollution in typical wheat fields.

The management of Cd pollution in acidic soils in the south of China has been carried out using mature technologies, such as passivation or foliar inhibitor [9]. The passivation strategy is to change the soil properties and bioavailability of Cd by adding alkaline substances, such as lime and calcium carbonate, to the soil. The technical principle of foliar-inhibitor strategy is that, spraying a barrier agent on crop leaves can change the distribution of Cd in plants, inhibit the transport of Cd from crop leaves to grains, and effectively reduce the accumulation of Cd in grains. But most of the Cd-contaminated wheat fields in the north of the country have alkaline soils, resulting in low effectiveness of Cd in the soil, so it is difficult to reduce the Cd content of wheat grain by adjusting pH value of the soil and using water management and other agronomic measures [10]. Moreover, high-efficiency passivation and foliar inhibitor suitable for Cd pollution in wheat fields with alkaline soil in the north have few materials, poor stability, and low barrier efficiency [11]. In addition, compared to maize, wheat has a stronger ability to accumulate Cd, which makes it more difficult to screen varieties with low-Cd accumulation [12], because studies have shown that the variation of Cd accumulation in the grain of different wheat varieties is smaller than that of maize varieties. For example, the variation of Cd accumulation among wheat was about 4 times [13], while the variation of Cd accumulation among maize was more than 10 times [14]. Last but not the least, the above methods of remediating Cd pollution cannot fundamentally reduce the Cd content in the soil “pool”.

Phytoremediation is currently the cleanest green and environmentally-friendly method for remediating Cd pollution in soils [15]. However, plants that hyper-accumulate Cd generally have long growth cycles, small biomass, and no advantage for remediation in alkaline soils with moderate-Cd concentrations [16]. According to the requirements to prevent cultivated land from being “non-grain” or “non-farm” in Decree 2021 No. 1 of the Ministry of Agriculture and Rural Affairs of China, seeking suitable low-Cd-accumulating plants to replace wheat cultivation for moderately Cd-contaminated wheat field is the best way to achieve safe production during remediation [17]. Therefore, in this study, green amaranth, oil sunflower, broomcorn, maize, and waxy maize were cultivated to study their Cd content and accumulation in different tissues, as well as to explore phytoremediation potential for Cd in soil, in order to provide theoretical and technical support for safe production during remediation in wheat fields with moderate Cd pollution.

## 2. Materials and Methods

### 2.1. Experimental Location

The experiment was conducted at the farm at Henan University of Science and Technology, which is located in Luoyang City (34°41′ N, 112°27′ E), Henan Province, China. The study area has a temperate-continental-monsoon climate with an average temperature of 12.2–24.6 °C, a frost-free period of more than 210 days, and annual precipitation and average humidity of 528–800 mm and 60–70%, respectively.

### 2.2. Experimental Material

The study plants included green amaranth (*Amaranthus hybridus* L.), maize (*Zea mays* L.), waxy maize (*Zea mays* L. var. ceratina Kulesh), broomcorn (*Sorghum bicolor* L.), and oil sunflower (*Helianthus annuus* L.). Cd-contaminated, calcareous soil was taken from the surface of wheat fields (0–20 cm) for potting. According to the soil environmental quality standard (GB15618-2018), the risk screening and intervention values for agricultural-soil contamination under the condition of soil pH > 7.5 are 0.6 and 4.0 mg∙kg^−1^, respectively. We referred to the annotation of the national soil pollution status survey bulletin [18] and used the screening value as the evaluation criterion to classify the soil as follows: heavily contamination: Cd content > 3.0 mg∙kg^−1^ (greater than 5 times the screening value); moderate contamination: 1.8 to 3.0 mg∙kg^−1^(between 3 and 5 times the screening value); light contamination: 1.2 to 1.8 mg∙kg^−1^ (between 2 and 3 times the screening value); and slight contamination: 0.6 to 1.2 mg∙kg^−1^ (between 1 and 2 times the screening value). Judging by the above criteria, the tested soil is moderately contaminated by Cd, and the basic physicochemical properties of soil were: pH of 8.08, organic matter of 30.11 g∙kg^−1^, total nitrogen of 0.821 g∙kg^−^^1^, available phosphorus of 22.10 mg∙kg^−^^1^, and available potassium of 112.32 mg∙kg^−^^1^. Total-Cd and available-Cd content were 2.48 and 0.94 mg∙kg^−1^, respectively.

### 2.3. Experimental Design

In this study, a plastic-pot experiment was conducted. The tested soil was air-dried and sieved through a 2-mm sieve, and 10 kg of soil was placed in each pot. The experimental design consisted of six treatments: blank control (CK, no plants), five kinds of plant treatment, including green amaranth, oil sunflower, broomcorn, maize, and waxy maize. According to the nutrient status of the soil, 2.60 g of CO(NH_2_)_2_, 4.20 g of Ca(H_2_PO_4_)_2_, and 1.30 g of KCl were applied to each pot. The soil was adjusted to 80% field-water-holding capacity, and one seedling of the above plants were transplanted in each pot when they grew to about 5 cm on 1 May 2021. Each treatment was replicated 6 times; these plastic pots were organized in complete-randomized design, and normal-watering management was carried out to keep the moisture level uniform in each pot.

### 2.4. Sample Collection and Analysis Methods

These plants were harvested on 19 August 2021, and harvested plant samples were separated according to the above- and below-ground parts. The plant tissues were washed with deionized water 2–3 times, oven dried (at 105 °C), homogenized by grinding in a metal-free, plastic mill, passed through a sieve of 0.85 mm of mesh size, and used for further chemical analysis. Soil samples were collected after plants were harvested and sieved through 0.85 mm and 0.12 mm of polyethylene mesh after air-drying. To air dry, soil samples were poured into a 2.3 cm-thick layer on a tray covered with white paper and placed in a cool, ventilated, and pollution-free environment. The soil was stirred with a wooden stick every morning and evening until the soil moisture content was less than 5%. The plant samples were digested by HNO_3_-HClO_4_, the soil samples were digested by HNO_3_-HClO_4_-HF, and the digested samples were measured by inductive-coupled-plasma-emission spectroscopy (Agilent 5110 ICP-OES). The soil Cd was extracted by the DTPA leaching method [19], and different types of speciation of Cd were extracted by referring to the BCR continuous extraction method [20], which divided the total Cd into acid extracted, reducible, oxidizable, and residual fractions. Other physicochemical properties of soil were determined with reference to the Methods of Agricultural Chemical Analysis of Soil [21]: The pH values of the soil samples (1:5 soil-to-water ratio, *w*/*v*) were measured by potentiometry using a pH meter (PHS-3E); the content of soil-organic matter was determined using the potassium-dichromate-digestion method; the total nitrogen of the soil was determined using the semi-micro-Kjeldahl method; the available phosphorus was measured with the sodium-bicarbonate method, and the available potassium was measured with the flame-photometry method.

### 2.5. Statistical Analysis

The data in this study were processed using Excel 2016 and statistically analyzed using SPSS Statistics 23’s the least significant difference (LSD) method to determine significant differences (*p* < 0.05). The following parameters were also introduced to characterize the enrichment characteristics and remediation potential of 5 plants in moderately Cd-contaminated soils [22,23,24,25]:

Bioaccumulation factor (BCF) = Cd concentration in plant tissues/Cd concentration in soil.(1)

Translocation factor (TF) = Cd content in stems, leaves, or grains/Cd content in roots.(2)

Cd accumulation of remediation plants = Cd content in plant aboveground × biomass of plant aboveground + Cd content in plant belowground × biomass of plant belowground.(3)

Extraction efficiency of remediation plants = plant Cd accumulation/(soil Cd content × soil mass) × 100%.(4)

Estimated restoration time required = soil mass × (soil Cd content − soil-risk-screening value)/Cd accumulation in plant aboveground.(5)

## 3. Results

### 3.1. Biomass of Remediation Plants

All five plants tested in this experiment grew normally in soil with a Cd content of 2.48 mg∙kg^−1^. As can be seen from Table 1, the dry weight of the five plants was 745.67, 96.81, 328.84, 339.82, and 328.09 g∙pot^−1^, respectively. Among them, the biomass of green amaranth was significantly higher than that of the other four plants, while the biomass of maize, broomcorn, and waxy maize was not significantly different, but was significantly higher than that of oil sunflower. The grain yield of oil sunflower, broomcorn, maize, and waxy maize was 42.71, 47.84, 165.69, and 166.30 g∙pot^−1^, respectively. Maize and waxy maize had significantly higher grain yield than oil sunflower and broomcorn.

### 3.2. The Speciation of Cd

The mobility, bioavailability, and eco-toxicity of Cd depend on its speciation more than its total content. The potential-bioactive speciation of Cd in soil is the acid-extracted fraction. In this study, the content of the total, acid-extracted, reducible, oxidizable, and residual fractions of Cd are shown in Table 2. Planting different plants had no significant impact on the change of soil-Cd speciation. The total, acid-extracted, reducible, oxidizable, and residual Cd content of different treatments ranged from 2.42 to 2.46, 0.43 to 0.49, 0.62 to 0.67, 0.50 to 0.56, and 0.80 to 0.84 mg∙kg^−1^, respectively. It can be seen that the residual Cd in soil was the dominant form, making up more than 32.5%, while the acid-extracted Cd was less than 20%. Compared with CK, short-period plant cultivation was not enough to change the Cd speciation in soil.

### 3.3. Characteristics of Cd Uptake and Enrichment by Plants

#### 3.3.1. Cd Content in Each Part of Plants

The content of acid-extracted Cd in tested soil was the lowest in all fractions (Table 2), but the roots of plants still absorbed Cd from the soil and transferred it to the stems, leaves, and other tissues of the plants via the xylem, phloem and other transport processes. Finally, Cd accumulated in the grain (Table 3). The Cd content in different parts of the green amaranth followed the order of root > leaf > stem; the Cd content in the different parts of oil sunflower was in the order of root > grain > leaf > stem > sunflower plate, while the Cd content in all the parts of broomcorn, maize, and waxy maize was in the order of root > leaf > stem > grain. Among them, the Cd content in roots, stems, and leaves of green amaranth was 0.88, 0.12, and 0.23 mg∙kg^−1^, respectively, lower than that in the same parts of other plants. The Cd content in the roots of broomcorn and oil sunflower was 3.57 and 3.30 mg∙kg^−1^, respectively, significantly higher than that in the green amaranth, maize, and waxy maize treatments. The Cd content in the leaves of maize and oil sunflower was 1.68 and 1.62 mg∙kg^−1^, respectively, which was significantly higher than that in the green amaranth, broomcorn, and waxy maize treatments. The Cd content in the stems and grains of oil sunflower was 1.32 and 2.29 mg∙kg^−1^, respectively, significantly higher than that in the stems and grains of other plants. In addition, Cd content in the grains of broomcorn, maize, and waxy maize was < 0.1 mg∙kg^−1^, lower than the national standard for contaminant limits in food (GB27; cereal grains and their products, except rice, are limited to 0.1 mg∙kg^−1^), and Cd content was the lowest in the grains of waxy maize, but the Cd content of oil sunflower grains exceeded the national standard for contaminant limits in food (GB2762-2017; for nuts and grains, the limit is 0.5 mg∙kg^−1^).

#### 3.3.2. Cd Enrichment Characteristics of Aboveground and Belowground Parts of Plants

Due to concern about food safety, it is important to understand the transfer of Cd from contaminated soil to plants. Table 4 shows the Cd-enrichment characteristics of different plants. The aboveground and total Cd content of plants displayed the order of oil sunflower > broomcorn > maize > waxy maize > green amaranth. The aboveground-, belowground-, and total-Cd contents of oil sunflower and broomcorn were significantly higher than those of green amaranth, maize, and waxy maize. The aboveground-Cd content of oil sunflower and broomcorn was 1.72 and 0.85 mg∙kg^−1^, respectively (12.29 and 6.07 times that of green amaranth); the belowground-Cd content was 3.30 and 3.57 mg∙kg^−1^, respectively (3.75 and 4.06 times that of green amaranth). The Cd content was significantly higher in the belowground parts of maize than that of green amaranth and waxy maize, while total-Cd content was significantly higher than that of green amaranth, but did not reach a significant level, compared with waxy maize. The Cd content of the belowground parts of maize was 1.90 mg∙kg^−1^, while that in the waxy maize was only 1.23 mg∙kg^−1^.

Plant enrichment refers to the ability of plants to absorb heavy metals from the surrounding soil environment and is generally expressed in terms of bioaccumulation factor (BCF), which is the ratio of the equilibrium concentration of a pollutant in an organism to the concentration of the same pollutant in its living environment [26], and is a measure of the tendency of heavy metals to accumulate in an organism. In this study, it can be seen that the aboveground parts of oil sunflower plants showed the highest bioaccumulation factor for Cd, which was 0.75. The bioaccumulation factors of the aboveground parts of green amaranth, broomcorn, maize, and waxy maize plants were less than 0.50 (0.06, 0.34, 0.19, and 0.12, respectively). The bioaccumulation factors of aboveground-Cd in different treatments were significantly different (Table 4).

Translocation factor (TF) refers to the ratio of Cd content in plant stems/leaves/grains to Cd content in roots, and a larger translocation factor indicates stronger transport capacity of the part for Cd [27]. Accumulation of Cd in the aboveground tissues depends not only on root uptake, but also on root-to-shoot translocation. As can be seen from Table 4, oil sunflower had the largest Cd-translocation factor of 0.52, which was significantly higher than that of the other four plants. The Cd-translocation factor of broomcorn, maize, and waxy maize was 0.24, 0.23 and 0.24, respectively, and there was no significant difference among these values. The Cd-translocation factor of green amaranth was the smallest among the five plants, which proved that the Cd-transport capacity of green amaranth was weak.

#### 3.3.3. Cd Accumulation in Each Part of Plants

The total accumulation of Cd by different plants is directly related to the remediation effects of plants on Cd-contaminated soil. The Cd-accumulation capacity of plant parts were different (Table 5): broomcorn and maize accumulated the most Cd, at 0.429 and 0.205 mg∙pot^−1^, respectively, and waxy maize accumulated the least, at 0.127 mg∙pot^−1^. The Cd accumulated by green amaranth and broomcorn was found mainly in the roots, while the Cd accumulated by oil sunflower was mainly in the grains, and the Cd accumulated by maize and waxy maize was mainly in the leaves. Analysis of variance showed that Cd accumulation was significantly higher in broomcorn than in the other four plants, especially in its roots and stems; maize had the higher Cd-accumulation ability than waxy maize. Nevertheless, Cd accumulation in the grains of broomcorn, maize, and waxy maize had no significance, compared to that in oil sunflower.

### 3.4. Phytoremediation Effects

The total-soil Cd decreased to different degrees in all treatments, compared to tested soil (Table 6). Among them, the reduction of total-soil Cd corresponding to the six treatments ranged from 0.18 to 0.54 mg∙pot^−1^, and the accumulation of Cd by plants from 0.13 to 0.43 mg∙pot^−1^. There was a difference between the Cd accumulation of remediation plants and the total-Cd reduction in soil. The reason is, on the one hand, due to Cd accumulation by plant; we calculated the extraction efficiency of remediation plants to be from 0.51% to 1.73%. On the other hand, it is due to leaching of available Cd, with rainfall and irrigation leading to partial-Cd loss; however, the specific form of leaching Cd needs to be further studied. If one of these plants is cultivated once per year, the restoration time required to remediate this soil to reach the risk-screening value of GB15618-2018 “Soil Environmental Quality Standard” for Cd contamination by green amaranth, oil sunflower, broomcorn, maize, and waxy maize will be at least 201, 119, 80, 142, and 214 years, respectively. In fact, if these remediation plants could be cultivated more than once per year, the Cd-contaminated soil may need less time to meet the soil-environmental-quality standard. 

## 4. Discussion

In China, due to the large population and limited cultivated land resources, the most reasonable way to remediate farmlands with moderate- or light-Cd pollution is to use the method of “production during remediation”. The most critical factor is to recommend suitable plants during the remediation process. In addition, in an unpublished investigation, we tested the plants used in the experiments described above in uncontaminated soil. We found that Cd accumulation at the tissues of maize, waxy maize, broomcorn, oil sunflower, and green amaranth in uncontaminated soil was 0.93–34.42%, 1.22–42.46%, 0.50–12.29%, 1.87–19.18%, and 3.13–23.12%, respectively, compared with Cd accumulation in the same tissues of the tested plants in contaminated soil. Thus, it can be seen that, in this study, the accumulation of Cd in all plant tissues in Cd-contaminated soil increased significantly, and different plants had different remediation effects on Cd. Therefore, it is necessary to evaluate the remediation potential of different plants for moderate-Cd-contaminated soil.

Green amaranth has the characteristics of fast growth, large biomass, easy cultivation, and strong adaptability. Study showed that the extraction rate of green amaranth in the soil with a Cd concentration of 5 mg∙kg^−1^ was 0.35%, higher than that of some Cd hyper-accumulators. The aboveground bioaccumulation and transport factors were 1.10 and 1.29 respectively, but its biomass was only 19.23 g∙pot^−1^, resulting in small Cd accumulation of 0.11 mg∙pot^−1^ [28]. He et al. (2018) investigated the variety-dependent ability of tolerance and accumulation of Cd in green amaranth [29]. In this experiment, although the Cd extraction efficiency was relatively low, which was 0.66%, the Cd accumulation reached 0.164 mg∙pot^−1^ due to its biomass, which suggests that the green amaranth we used mainly depends on huge biomass to increase Cd accumulation in moderate-Cd-contaminated soil. 

Oil sunflower is a major crop that has a strong enrichment ability of Cd. In field experiments, the amount of Cd accumulated by oil sunflower was significantly higher than that of rape [30]. Yang et al. (2017) found that when 13 high-Cd-accumulation varieties of oil sunflower were cultivated in the soil with Cd content of 0.62 and 2.08, respectively, the Cd content in the grains reached 4.03–5.84 and 4.71–7.03 mg∙kg^−1^, respectively [31]. In this experiment, the Cd content of oil-sunflower grains and aboveground-bioaccumulation factor were 2.29 mg∙kg^−1^ and 0.75, respectively, much higher than those of other plants in this study; however, its biomass was only 96.81 g∙pot^−1^, resulting in a small accumulation of 0.176 mg∙pot^−1^ of Cd. Therefore, if the biomass of oil sunflower can be increased and the aboveground part of oil sunflower can be removed from the soil, it will serve as another effective restoration strategy.

Broomcorn is characterized by its large biomass, short growth cycle, developed root system, and strong stress resilience. Metwali et al. (2013) compared the tolerance of broomcorn, maize, and wheat to Cd, and showed that broomcorn had the strongest tolerance and absorbed the highest amount of Cd [32]. Similar Cd-accumulation results were obtained in this experiment where the Cd accumulation in broomcorn reached 0.429 mg∙pot^−1^, which was 3.37 and 2.09 times that of waxy maize and maize. Zheng et al. (2018) found that Cd accumulation in the aboveground part of two kinds of broomcorn exceeded 65 g∙ha^−1^, which was 2–3 times higher than that of sweet broomcorn [33]. This is because sweet broomcorn, which is not a hyper-accumulator plant, mainly accumulates Cd in its roots, and only a small amount is transferred to the aboveground part [34]. It can be seen that broomcorn is more effective than sweet broomcorn in remediating Cd-contaminated soil. In this study, when the broomcorn variety with high stems was selected, which can extend the distance of Cd transportation through its xylem, Cd accumulation was higher than other plants, but the Cd content of its grain was only 0.002 mg∙kg^−1^, concluding that this kind of broomcorn can ensure safe production during remediation.

Maize, as an important food and feed crop in China, has weaker Cd-transport capacity, compared to rice, wheat, and soybean. Generally, the stem-leaf/root translocation factor is <1 and grain/stem-leaf translocation factor is <0.5 [35]. For example, Yang et al. (2014) found that the Cd-transport coefficient in the upper part of different maize varieties ranged from 0.04 to 0.45 [36]. Compared to rice, wheat, and vegetables, maize is an ideal plant for remediation of moderately-Cd-contaminated soils due to its larger biomass and lower Cd content in the grains. Study showed that Cd was mainly retained in maize roots and a small quantity was translocated to its shoot [37]. In maize roots, Cd was mainly accumulated in the rhizodermis and the cortex of the outer-root tissues [38]. In addition, maize has a lesser Cd-uptake ability because the expression and functionality of maize Nramp5 are lower, and its protein has a lower Cd-transport activity [39]. Waxy maize can also be used as a cultivating crop for arable land with moderate Cd pollution. Research showed that when Cd content in the soil increased from 1 to 3 mg∙kg^−1^, the Cd content in the roots was higher in maize than in waxy maize, but the Cd content in stems, leaves, and grains was significantly lower in maize than in waxy maize, which shows that it is relatively safer to cultivate maize than waxy maize on Cd-contaminated soil [40], but research results were inconclusive when cultivating maize and waxy maize in soil with a Cd content of 2.2 mg∙kg^−1^ [41]. In this study, the Cd content was significantly higher in maize roots and leaves than in waxy maize roots, and was also slightly higher in maize grains than in waxy maize, but it did not reach a significant level. At present, there are no clear standards to distinguish and define maize varieties with high- and low-Cd enrichment, and the above results may be due to genetic factors of the crop itself, such as different genotypes of maize with different Kjeldahl-band structures, and types and content of compounds inside the xylem and phloem [42]. Since remediation of Cd in soil by food crops is mostly concentrated in hydroponics and pot experiments, and involves few field experiments [43], it is necessary to further verify the production effect in combination with field experiments, and improve the efficiency of phytoremediation through strengthening measures.

## 5. Conclusions

In the process of remediating moderately Cd-contaminated soil by five plants, the Cd content in grains of broomcorn, maize, and waxy maize was less than 0.1 mg∙kg^−1^, which is lower than the national food safety standard regarding pollutant limits in food (GB2762-2017), which demonstrates safe food production. The biomass of green amaranth was much larger than that of the other four plants, but its extraction efficiency and accumulation of Cd were low; the Cd-enrichment capacity of oil sunflower was strong, but its biomass was small and the Cd content in grains seriously exceeded the standard. The Cd accumulation and extraction efficiency of broomcorn were higher than those of the other four plants. For the purpose of our study, which is safe food production during remediation, broomcorn will be more conducive to production during restoration of moderately Cd-contaminated soil. 

## Figures and Tables

**Table 1:** The biomass of each part of different plants (g∙pot^−1^).

Treatments	Root	Stem	Leaf	Grain	Oil Sunflower Plate	Dry Weight of Plants
Green amaranth	81.17 ± 12.21 a	521.53 ± 112.23 a	142.97 ± 49.17 a	——	——	745.67 ± 123.97 a
Oil sunflower	5.67 ± 1.68 d	20.45 ± 2.15 c	11.82 ± 0.69 c	42.71 ± 12.67 b	16.16 ± 1.11	96.81 ± 15.43 c
Broomcorn	54.59 ± 2.72 b	148.85 ± 11.64 b	77.56 ± 6.78 b	47.84 ± 9.61 b	——	328.84 ± 29.12 b
Maize	38.28 ± 1.15 c	66.77 ± 4.33 bc	69.08 ± 1.99 b	165.69 ± 0.89 a	——	339.82 ± 4.28 b
Waxy maize	32.09 ± 0.70 c	55.00 ± 3.70 bc	74.70 ± 2.98 b	166.30 ± 1.56 a	——	328.09 ± 7.07 b

Note: The data are mean ± standard deviation (n = 6), different lowercase letters in the same column indicate significant difference between treatments (*p* < 0.05), and dash (——) represents absence of the site.

**Table 2:** The speciation of Cd in soil after restoration by different plants (mg∙kg^−1^).

Treatments	Total	Acid Extracted	Reducible	Oxidizable	Residual
CK	2.46 ± 0.07 a	0.44 ± 0.14 a	0.65 ± 0.15 a	0.53 ± 0.19 a	0.84 ± 0.14 a
Green amaranth	2.45 ± 0.02 a	0.45 ± 0.13 a	0.67 ± 0.12 a	0.52 ± 0.16 a	0.81 ± 0.03 a
Oil sunflower	2.44 ± 0.10 a	0.44 ± 0.20 a	0.65 ± 0.16 a	0.55 ± 0.23 a	0.80 ± 0.04 a
Broomcorn	2.42 ± 0.01 a	0.43 ± 0.10 a	0.62 ± 0.10 a	0.56 ± 0.25 a	0.81 ± 0.18 a
Maize	2.44 ± 0.15 a	0.48 ± 0.14 a	0.66 ± 0.08 a	0.50 ± 0.12 a	0.80 ± 0.13 a
Waxy maize	2.46 ± 0.05 a	0.49 ± 0.10 a	0.66 ± 0.08 a	0.51 ± 0.12 a	0.80 ± 0.07 a

Note: The data are mean ± standard deviation (n = 6), different lowercase letters in the same column indicate significant difference between treatments (*p* < 0.05).

**Table 3:** Cd content in each part of different plants (mg∙kg^−1^).

Treatments	Root	Stem	Leaf	Grain	Oil-Sunflower Plate
Green amaranth	0.88 ± 0.26 c	0.12 ± 0.02 c	0.23 ± 0.02 c	——	——
Oil sunflower	3.30 ± 0.51 a	1.32 ± 0.07 a	1.62 ± 0.06 a	2.29 ± 0.51 a	0.78 ± 0.07
Broomcorn	3.57 ± 0.25 a	0.97 ± 0.03 b	1.11 ± 0.08 b	0.05 ± 0.00 b	——
Maize	1.90 ± 0.33 b	0.18 ± 0.07 c	1.68 ± 0.31 a	0.03 ± 0.01 b	——
Waxy maize	1.23 ± 0.14 c	0.19 ± 0.01 c	1.00 ± 0.03 b	0.02 ± 0.00 b	——

Note: The data are mean ± standard deviation (n = 6), different lowercase letters in the same column indicate significant difference between treatments (*p* < 0.05), and dash (——) represents absence of the site.

**Table 4:** Cd-enrichment characteristics of aboveground- and belowground-parts of plants.

Treatments	Content of Aboveground Part (mg∙kg^−1^)	Content of Belowground Part (mg∙kg^−1^)	Total Content (mg∙kg^−1^)	BCF of Aboveground	TF of Aboveground
Green amaranth	0.14 ± 0.01 c	0.88 ± 0.26 c	0.22 ± 0.03 d	0.06 ± 0.01 e	0.17 ± 0.06 b
Oil sunflower	1.72 ± 0.29 a	3.30 ± 0.51 a	1.81 ± 0.27 a	0.75 ± 0.08 a	0.52 ± 0.06 a
Broomcorn	0.85 ± 0.04 b	3.57 ± 0.25 a	1.31 ± 0.05 b	0.34 ± 0.02 b	0.24 ± 0.02 b
Maize	0.44 ± 0.07 c	1.90 ± 0.33 b	0.60 ± 0.09 c	0.19 ± 0.01 c	0.23 ± 0.03 b
Waxy maize	0.30 ± 0.01 c	1.23 ± 0.14 c	0.39 ± 0.02 cd	0.12 ± 0.00 d	0.24 ± 0.02 b

Note: The data are mean ± standard deviation (n = 6), different lowercase letters in the same column indicate significant difference between treatments (*p* < 0.05).

**Table 5:** Cd accumulation in each part of different plants (mg∙pot^−1^).

Treatments	Root	Stem	Leaf	Grain	Oil Sunflower Plate	Total Plant
Green amaranth	0.070 ± 0.021 b	0.061 ± 0.010 b	0.032 ± 0.009 c	——	——	0.164 ± 0.025 bc
Oil sunflower	0.019 ± 0.006 c	0.027 ± 0.004 c	0.019 ± 0.001 c	0.099 ± 0.038 a	0.013 ± 0.002	0.176 ± 0.041 bc
Broomcorn	0.195 ± 0.020 a	0.145 ± 0.011 a	0.086 ± 0.008 b	0.002 ± 0.000 b	——	0.429 ± 0.036 a
Maize	0.073 ± 0.012 b	0.012 ± 0.005 d	0.115 ± 0.019 a	0.005 ± 0.001 b	——	0.205 ± 0.030 b
Waxy maize	0.039 ± 0.005 c	0.011 ± 0.000 d	0.074 ± 0.003 b	0.003 ± 0.000 b	——	0.127 ± 0.005 c

Note: The data are mean ± standard deviation (n = 6), different lowercase letters in the same column indicate significant difference between treatments (*p* < 0.05), and dash (——) represents absence of the site.

**Table 6:** Phytoremediation effects of different plants.

Treatments	Total Cd (mg∙pot^−1^)	Total-Cd Reduction (mg∙pot^−1^)	Cd Accumulation of Remediation Plants (mg∙pot^−1^)	Extraction Efficiency of Remediation Plants (%)	Estimated Restoration Time Required (years)
Tested soil	24.76	——	——	——	——
CK	24.58	0.18	——	——	——
Green amaranth	24.53	0.23	0.16	0.66%	201
Oil sunflower	24.42	0.34	0.18	0.71%	119
Broomcorn	24.22	0.54	0.43	1.73%	80
Maize	24.41	0.35	0.20	0.83%	142
Waxy maize	24.57	0.19	0.13	0.51%	214

Note: The data are mean (n = 6). Total Cd reduction = Total Cd in tested soil − Total Cd in treatments, and dash (——) represents absence of the site.

## Data Availability

This study includes all supporting data, which can be obtained from the corresponding authors upon request.
